# Flame Retardancy and Mechanical Properties of Melt-Spun PA66 Fibers Prepared by End-Group Blocking Technology

**DOI:** 10.3390/polym15051183

**Published:** 2023-02-26

**Authors:** Yanpeng Wu, Tonghui Yang, Yongchang Cheng, Tao Huang, Bin Yu, Qilin Wu, Meifang Zhu, Hao Yu

**Affiliations:** State Key Laboratory for Modification of Chemical Fibers and Polymer Materials, College of Materials Science and Engineering, Donghua University, Shanghai 201620, China

**Keywords:** PA66, Di−PE, flame retardance, melt spinning, flame retardant mechanism

## Abstract

Preparing flame-retardant polyamide 66 (PA66) fibers through melt spinning remains one of the biggest challenges nowadays. In this work, dipentaerythritol (Di−PE), an eco-friendly flame retardant, was blended into PA66 to prepare PA66/Di−PE composites and fibers. It was confirmed that Di−PE could significantly improve the flame-retardant properties of PA66 by blocking the terminal carboxyl groups, which was conducive to the formation of a continuous and compact char layer and the reduced production of combustible gas. The combustion results of the composites showed that the limiting oxygen index (LOI) increased from 23.5% to 29.4%, and underwriter laboratories 94 (UL-94) passed the V-0 grade. The peak of heat release rate (PHRR), total heat release (THR), and total smoke production (TSP) decreased by 47.3%, 47.8%, and 44.8%, respectively, for the PA66/6 wt% Di−PE composite compared to those recorded for pure PA66. More importantly, the PA66/Di−PE composites possessed excellent spinnability. The prepared fibers still had good mechanical properties (tensile strength: 5.7 ± 0.2 cN/dtex), while maintaining good flame-retardant properties (LOI: 28.6%). This study provides an outstanding industrial production strategy for fabricating flame-retardant PA66 plastics and fibers.

## 1. Introduction

A survey from the Fire and Rescue Department Ministry of Emergency Management of China reports that 1.324 million fire accidents, 11,634 deaths, and 6738 injuries resulting from residential fires occurred nationwide over the period of 2012–2022. Polymeric materials are widely used in everyday day life, which increases the fire hazards [1]. The fire problem caused by textiles has always plagued people all over the world and seriously threatens people’s lives and property, public safety, and social development. Polyamide 66 (PA66), also known as nylon 66, is a thermoplastic textile material used in many fields including apparels and industrial textiles due to its excellent properties such as good mechanical behavior, low cost, resistance to shrinkage and pleasant aesthetics [2,3]. However, PA66 is flammable and also present a melt dripping problem during combustion, which limits its wide application [4]. Therefore, it is necessary and remains a huge challenge to prepare PA66 fabrics with excellent flame retardance properties.

Conventional approaches that can be considered to improve the flame retardance property of PA66 fabric include post-finishing, copolymerization, and blending. Among them, the flame-retardant finishing of PA66 fabric is the most widely used due to its simple processing [5,6,7]. Thiourea–formaldehyde is one of the most successful durable flame-retardance finishing systems for polyamide fabrics, but it releases formaldehyde during processing and use, which has known toxic effects on the environment and human health [8,9]. Although some research teams have developed relatively green flame-retardant finishing systems, their flame-retardant efficiency and durability appeared reduced [10,11]. More importantly, the biggest common problem of the post-finishing method is that the wear comfort of the fabric, including air permeability, hygroscopicity, flexibility, etc., is often sacrificed during the finishing process.

Compared with post-finishing, copolymerization can impart PA66 intrinsic flame-retardancy and enhanced durability [12,13,14,15,16]. Li et al. [17] prepared flame-retardant PA66 composites by condensation polymerization of a PA66 salt with 5.5 wt% of a 9,10-dihydro-9-oxa-10-phosphaphenanthrene-10-oxide (DOPO)-based flame retardant. The limiting oxygen index (LOI) value and underwriter laboratories 94 (UL-94) of the PA66 composites reached 32.9% and V-0 rating, respectively. It is worth noting that, although the authors prepared PA66 fibers by using the flame-retardant PA66 composites through melt spinning, unfortunately, the fiber strength was decreased by 37.4% compared with that of bare PA66, and more importantly, the flame-retardant performance of the composite fiber was not evaluated. As for the blending method, it has more advantages than the copolymerization method because of the simple preparation required and the easiness to scale up production to industrial levels. However, as far as the authors know, there are few reports about flame-retardant PA66 fibers or fabrics which are prepared by the blending method, and almost all of them are plastic products [18,19]. The reasons behind this are as follows: (1) the addition of flame retardants often results in poor spinnability of PA66 and even failure to spin due to the poor dispersion and compatibility; (2) there is a lack of high-performance flame retardants suitable for the melt spinning of nylon 66, which needs to meet lack of toxicity, high thermal stability, and good spinnability and to achieve high flame retardant efficiency with the addition of a low amount.

Herein, a series of PA66 flame retardant composites were prepared by the simple blending method using dipentaerythritol (Di−PE) as a flame retardant. The effect of different mass ratios of Di−PE on the flame retardancy of the PA66/Di−PE composites was investigated by limited oxygen index, underwriter laboratories 94, and cone calorimeter tests, and the possible flame retardancy mechanism was also explored. More importantly, PA66 fibers with excellent flame retardance performance were finally obtained via melt spinning and their mechanical properties were also evaluated.

## 2. Materials and Methods

### 2.1. Materials and Machining Equipment

PA66 was purchased from Shen Ma Industry Co., Ltd., China. Di−PE (90%) was purchased from Shanghai Aladdin Bio-Chem Technology Co., Ltd., Shanghai, China. The materials were used as received. The twin-screw extruder used (D: 21.7 mm, L/D: 40, model, SHJ-20) was purchased from Nanjing GIANT Machinery Co., Ltd., Nanjing, China. The vertical injection molding machine (TY-200) was purchased from TAYU Machinery Co., Ltd., China. The two-component composite spinning machine (Abe Φ25∗2, D = 25 mm, L/D = 28) was purchased from Abe Corporation, Japan.

### 2.2. Preparation of Flame-Retardant PA66 Composites

Di−PE and PA66 were dried overnight in a vacuum oven at 110 °C. The flame retardant PA66 composites were obtained by extruding the mixtures of PA66 and Di−PE in a twin-screw extruder. The temperature of the extruder was maintained at 230 °C, 245 °C, 260 °C, 270 °C, 265 °C, and 265 °C from hopper to die. The dried pellets of the composites were molded by the vertical injection molding machine at a temperature in three heating zones (240 °C, 270 °C, and 267 °C).

### 2.3. Melt Spinning of PA66 and PA66/Di−PE Composites

The pellets of PA66 and PA66/Di−PE composites were melt-spun with the two-component composite spinning machine. The main spinning parameters are summarized in Table 1.

### 2.4. Characterization

#### 2.4.1. Limiting Oxygen Index

The LOI test was determined on an oxygen index meter (TTech-GBT2406-2, TES Tech Instrument Technologies Co., Ltd., Suzhou, China) with 80 mm × 10 mm × 4.0 mm specimens according to GB/T 2406.2-2009.

#### 2.4.2. UL-94 Vertical Burning

The UL-94 tests were performed based on a horizontal and vertical burning test instrument (ZR-02 vertical burning test instrument, Qingdao Shanfang Instrument Co., Ltd., Qingdao, China) according to the GBT2408-2008 testing procedure, using samples with dimensions of 127 mm × 12.7 mm × 3.2 mm.

#### 2.4.3. Fourier Transform Infrared Spectroscopy (FTIR)

The ATR-FTIR spectra of the PA66/Di−PE composites and char residues were obtained with a Nicolet 6700 FTIR instrument (Thermo Fisher Scientific, Waltham, Massachusetts, USA). The wavenumber range was set from 4000 to 600 cm^−1^.

#### 2.4.4. Thermogravimetry Analysis

The thermal properties of the samples were studied with a thermogravimeter (TG 209 F1, Netzsch Netzsch, Selb, Germany). Fir this, 5–10 mg samples were heated at 20 °C/min from 25 to 700 °C under air atmosphere.

Differential scanning calorimetry (DSC) was performed on a Netzsch DSC 204 F1 (Selb, Germany), with a heating rate of 20 °C/min up to 280 °C under nitrogen and held for 2 min to completely remove the previous thermal history. Then, the samples were cooled down to 25 °C and finally heated to 280 °C at the cooling and heating rates of 10 °C/min.

#### 2.4.5. Cone Calorimeter Test

The combustion performance was evaluated with a cone calorimeter (i-Cone, Fire Testing Technology Ltd., East Grinstead, UK) in accordance with ISO 5660-1 standard, using specimen with dimensions of 100 mm × 100 mm × 3 mm with 50 kW/m^2^.

#### 2.4.6. Scanning Electron Microscope (SEM) Analysis

The residual chars were obtained in a muffle furnace at 500 °C, then observed by a scanning electron microscope (FlexSEM 1000II, Hitachi, Tokyo, Japan) at the accelerating voltage of 10 kV.

#### 2.4.7. Raman Analysis

The Raman spectra of PA66 and the PA66/Di−PE composites residual chars were obtained with a laser Raman spectrometer (Renishaw inVia Reflex, Renishaw Co., Miskin, UK) using a 532 nm helium–neon laser line focused on a micrometer spot on the sample surface and scanning in the 2000–800 cm^−1^ region.

#### 2.4.8. X-ray Photoelectron Spectroscopy

The XPS data were carried obtained with an Escalab 250Xi electron analyzer (Thermo Fisher Scientific Co., Waltham, MA, USA).

#### 2.4.9. Thermogravimetry–Fourier Transform Infrared Spectrometry

The TG–FTIR was conducted on a thermogravimeter instrument (TG 209 F1, Netzsch Netzsch, Selb, Germany) with a Fourier transform infrared spectrometer (Tensor 27, Bruker Optics, Ettlingen, Germany). About 10 mg of the samples was heated at 20 °C/min from 25 to 800 °C under air atmosphere. The pyrolysis gaseous products were tunneled into the FTIR device and scanned in the range from 4000 to 600 cm^−1^.

## 3. Results

### 3.1. Thermal Stability of the PA66/Di−PE Composites

**Figure 1:**
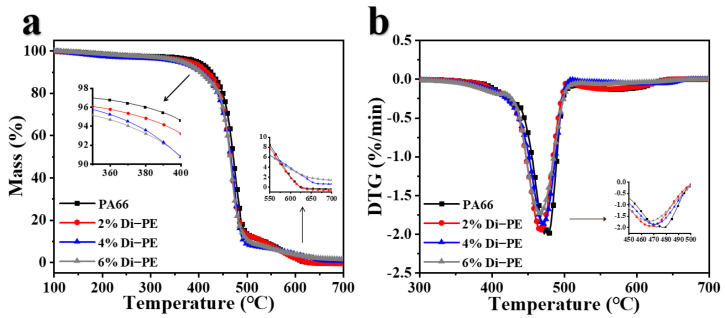
TG (**a**) and DTG (**b**) curves for PA66 and the PA66/Di−PE composites in air conditions.

Thermogravimetric analysis (TGA) and derivative thermogravimetry (DTG) curves in the air atmosphere were utilized to assess the thermal-oxidative degradation behavior of PA66 and its composites (Figure 1). The summary of the results is presented in Table 2. T_5%_ is the temperature corresponding to a 5 wt% weight loss and is indispensable to evaluate the thermal decomposition behavior of a polymer in the initial stage. The pure PA66 presented almost no char residue above 600 °C, and the T_5%_ and T_max_ (the temperature at the maximum weight loss rate) were about 396.3 °C and 477.7 °C, respectively.

It is obvious that in the presence of Di−PE, both the T_5%_ and the T_max_ of the PA66/Di−PE composites decreased, while the char yields of the PA66/4 wt% Di−PE and PA66/6 wt% Di−PE composites at 600 °C or 700 °C were much higher than that of pure PA66. In order to more intuitively verify that Di−PE promoted char formation from PA66, PA66 and PA66/6 wt% Di−PE were placed in muffle furnaces at different temperatures, and it was found that PA66/6 wt% Di−PE formed carbon layers earlier than PA66 at the same high temperature (Figure S1). Such a phenomenon confirmed that the presence of Di−PE promoted the decomposition and the charring process of the PA66/Di−PE composites in air atmosphere, which might contribute to the flame-retardant performance.

### 3.2. Flame-Retardant and Combustion Behavior

The LOI and UL-94 vertical burning tests were used to study the flame-retardant performance of the PA66/Di−PE composites. The corresponding results are listed in Table 3. The pure PA66 can be ignited and burnt with dripping, showing flammable characteristics. For the PA66/Di−PE composite, the LOI value increased with the Di−PE content. The LOI value of the PA66/Di−PE composite with a relatively low loading of 2 wt% could reach 28.80%, but the UL-94 rating was still V-2. Interestingly, a maximum LOI value of 29.4% and a UL-94 V-0 rating could be reached by further increasing the Di−PE content to 6 wt%. Moreover, the color of the char layer, which was formed in the PA66/Di−PE after the LOI test gradually, turned black with the increasing content of Di−PE, indicating that a higher content of the char layer and a higher degree of carbonization were obtained (Figure S2). These results showed that Di−PE was effective in improving the flame retardance of PA66.

**Figure 2:**
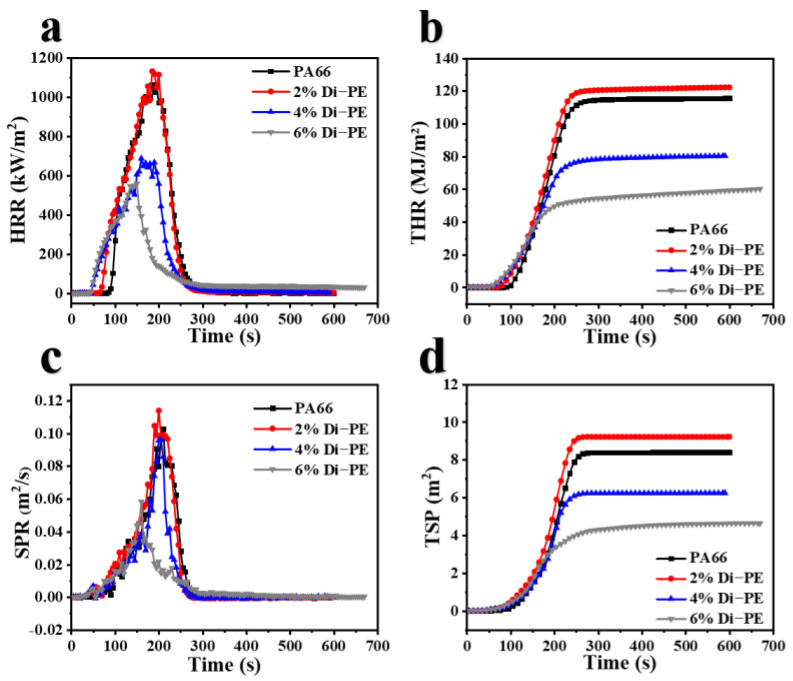
Curves of HRR (**a**); THR (**b**); SPR (**c**); and TSP (**d**).

In order to further evaluate the combustion process of the PA66/Di−PE composites in real combustion situations, the cone calorimetry test (CCT) was implemented. The CCT is a significant method to evaluate the fire behavior of polymers. It simulates fire hazard conditions and provides a series of key flammability parameters such as heat release rate (HRR), peak of heat release rate (PHRR), total heat release (THR), effective heat of combustion (EHC), mass loss rate (MLR), total smoke production (TSP), peak of smoke production rate (PSPR), average carbon monoxide yield (av-COY), average carbon dioxide yield (av−CO_2_Y) [20,21], and non-dimensional flame retardancy index (FRI) [22,23]. In general, lower values of PHRR, av-HRR, and THR indicate a better flame-retardant effect and are used to evaluate the fire safety of the materials in a real fire. It can be seen in Figure 2a,b and Table 4 that the PHRR and THR of PA66/2 wt% Di−PE were slightly higher than those of pure PA66. This was caused by the violent thermal degradation of the composite at a high temperature and the insufficient flame-retardant properties of the PA66/2 wt% Di−PE composite. In addition, with the increasing content of Di−PE, the values of PHRR, av-HRR, and THR decreased. Specifically, the PHRR, av-HRR, and THR of PA66/6 wt% Di−PE sharply decreased to 558.7 kW/m^2^, 112.8 kW/m^2^, and 60.1 MJ/m^2^, which represent a reduction by 47.3%, 68.6%, and 47.8%, respectively, compared to pure PA66. Figure 2c,d also presents the SPR and TSP curves of the PA66/Di−PE composites, which are considered other important parameters related to the hazard that threatens the life and environment in a real fire. Similar to the THR and HRR curves, the PA66/Di−PE composites showed significantly reduced values of TSP and SPR, while the Di−PE content was greater than or equal to 4 wt%, as compared to that in pure PA66. The PSPR and TSP of pure PA66/6 wt% decreased by 43.7% and 44.8%, respectively. Comprehensively, all the above data and curves (HRR, THR, SPR, and TSP) indicated the marked improvement of the flame retardancy and smoke suppression performance of PA66 with the incorporation of Di−PE. It acquired satisfactory flame-retardant properties, comparing with other works in the Table S1 (Supplementary Materials).

Additionally, the flame retardancy index (FRI), a non-dimensional criterion to evaluate the flame-retardant properties of composites, was calculated according to the following formula:

$$\begin{array}{c} \mathrm{FRI}=\frac{{\left[\mathrm{THR}\times \left(\frac{\mathrm{PHRR}}{\mathrm{TTI}}\right)\right]}_{\mathrm{Neat}\;\mathrm{polymer}}}{{\left[\mathrm{THR}\times \left(\frac{\mathrm{PHRR}}{\mathrm{TTI}}\right)\right]}_{\mathrm{Composite}}} \end{array}$$

The FRI is an important parameter for evaluating the flame-retardant properties of a composite. More specifically, the FRI is classified as Poor (FRI ≤ 1), Good (1 < FRI ≤ 10), and Excellent (FRI > 10) [24]. With the incorporation of Di−PE, the FRI of the PA66/Di−PE composites improved when the concentration of Di−PE increased. As shown in Table 4, when the concentration of Di−PE in PA66 was 4 wt% and 6 wt%, the FRI of PA66/4 wt% Di−PE and PA66/6 wt% Di−PE composites reached 1.53 and 2.42, respectively, which correspond to a “Good” level of fire safety. The higher FRI for the PA66/4 wt% Di−PE and PA66/6 wt% Di−PE composites proved their remarkable fire retardancy ability.

### 3.3. Analysis of the Flame-Retardant Mechanism

**Figure 3:**
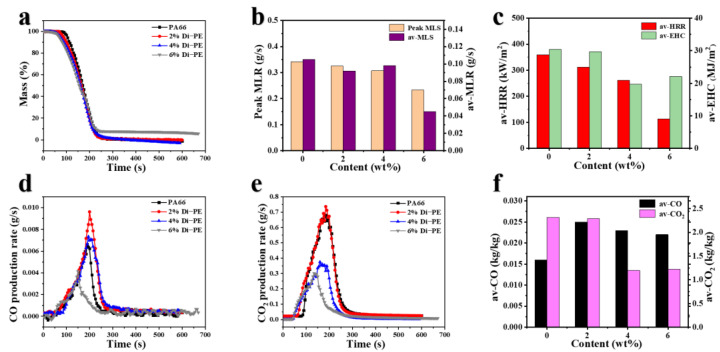
Mass (**a**), peak MLR, and av−MLR (**b**); av-HRR and av-EHC (**c**); CO production rate (**d**); CO_2_ production rate (**e**); av-CO-CO_2_ (**f**) for PA66 and the PA66/Di−PE composites.

The Cone test not only describes the flame retardance of samples but also can be used to study flame-retardant mechanisms [25]. The TTI of the PA66/Di−PE composites decreased compared to that of pure PA66, indicating that Di−PE promoted the catalytic decomposition of PA66 to form char layers, which provided an effective shield protection to restrain the intensity of smoke emission, thus reducing the SPR and TSP. The mass loss curves (Figure 3a) also confirmed that the thermal decomposition time of PA66/Di−PE was shorter than that of pure PA66, which is in accordance with the TG result, while the peak MLR and av-MLR values of the PA66/Di−PE composites were lower than those of pure PA66, thus resulting in increased amounts of char residuals, indicating that Di−PE played a flame retardant role in the condensed phase.

EHC is an important parameter to demonstrate the degree of combustion of volatile gas in the gas phase [26]. Figure 3c shows that the EHC of the PA66/Di−PE composites was lower compared with that of PA66, indicating that the incorporation of Di−PE led to incomplete combustion in the gas phase, which was also confirmed by the av-COY and av-CO_2_Y shown in Figure 3d–f. Clearly, the av-COY increased, whereas the av-CO_2_Y decreased with the increase of Di−PE loading. These results showed that Di−PE also played a flame-retardant role in the gas phase.

**Figure 4:**
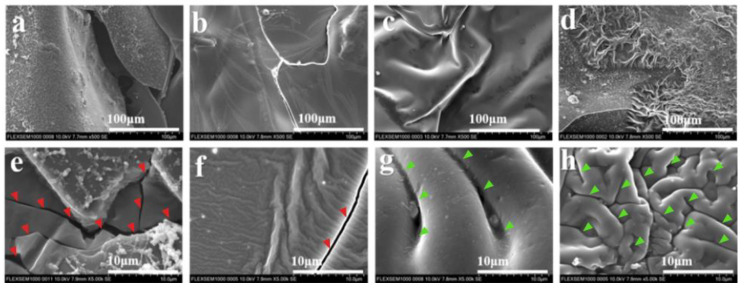
SEM images of the carbon layer on the surface of PA66 (**a**,**e**); PA66/2 wt% Di−PE (**b**,**f**); PA66/4 wt% Di−PE (**c**,**g**); and PA66/6 wt% Di−PE (**d**,**h**) at different magnifications. The red triangle refers to the fractured layer, and the green triangle refers to the dense layer.

In order to fully understand the flame retardance mechanism of the PA66/Di−PE composites, ATR-FTIR, SEM, Raman spectroscopy, XPS, and TG-IR were utilized to investigate the properties of the char residues and the volatile components. Figure 4 shows the microstructures of the residual chars for pure PA66 and the PA66/Di−PE composites. Clearly, it can be observed that the char layer of pure PA66 was fragile with many cracks and holes (Figure 4a,e, the red triangle), which would provide pathways for inflammable volatile products to mix with oxygen, then leading to the flame propagation. Therefore, the char layer cannot provide good flame protection for the underlying material from further burning. Interestingly, the char layers of PA66/2 wt% Di−PE only showed a few microcracks and were more continuous compared with those of pure PA66 (Figure 4b,f, the red triangle). Further increasing the content of Di−PE to 4 wt% (Figure 4c,g, green triangle) and 6 wt% (Figure 4d,h, green triangle), uniform and compact char layers with wrinkled morphologies formed instead of cracks and holes. As a result, the outstanding barrier effect of this kind of char layer can effectively prevent the transmission of heat, oxygen, and combustible gases, thus protecting the internal PA66 matrix from flame.

**Figure 5:**
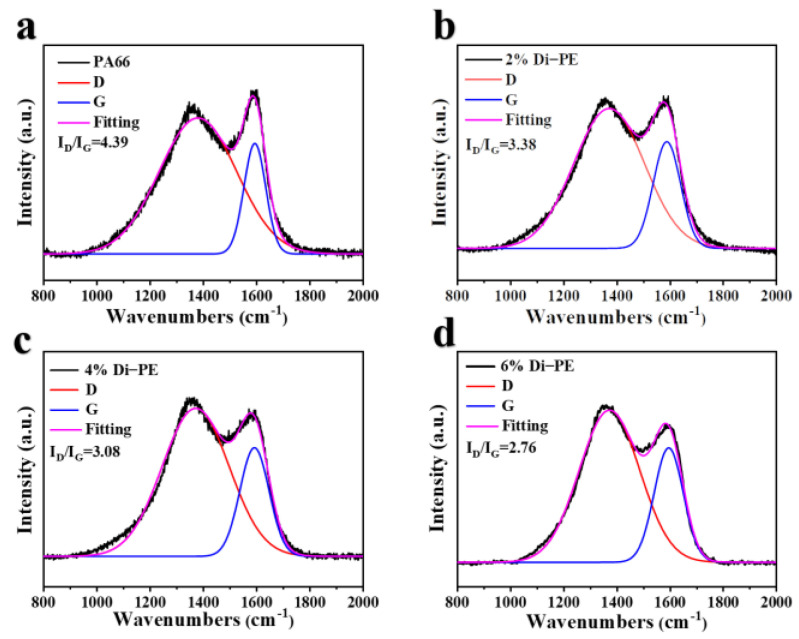
Raman spectra of char residues for PA66 (**a**); PA66/2 wt% Di−PE (**b**); PA66/4% Di−PE (**c**); and PA66/6% Di−PE (**d**); the fitting equation was Gaussian.

Moreover, besides the morphology of the char, the graphitization degree of the char also played an important role in improving the flame-retardant properties. The higher the graphitization degree in the char structure, the better the protection against thermal degradation of the material. Thus, Raman spectroscopy was applied to analyze the carbonaceous structures and graphitization of residual char (Figure 5). The graphitization degree of residual char was assessed by the relative intensity ratio of the D and G bands (I_D_/I_G_); the fitting equation is Gaussian, and the lower the I_D_/I_G_ value, the higher the graphitization degree [27,28,29]. It is obvious that the I_D_/I_G_ value decreased with the increasing content of Di−PE. Therefore, both the microstructure and the graphitization degree of the char residuals further indicated that Di−PE played a positive effect on enhancing the flame retardance in the condensed phase, which could promote the formation of a compact and continuous char layer with a high graphitization degree, thus protecting the matrix effectively.

**Figure 6:**
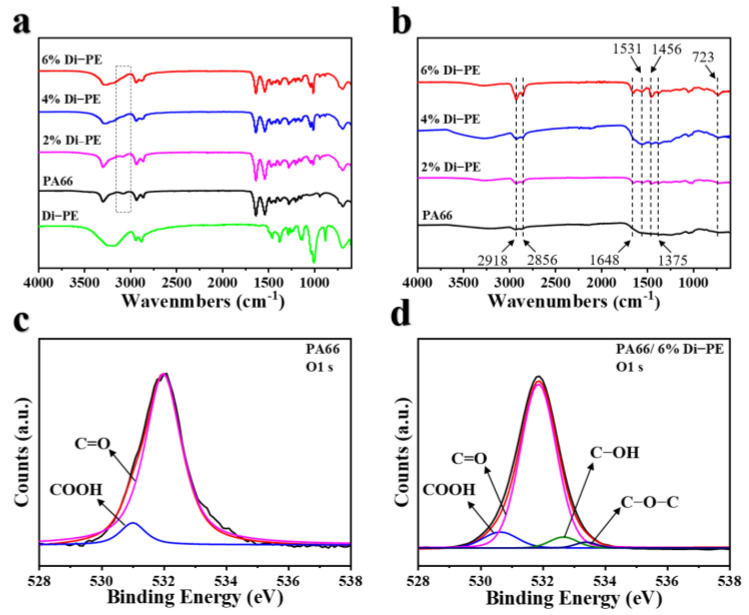
ATR-FTIR analyses of PA66 and PA66/Di−PE composites (**a**); FTIR spectra of the PA66/Di−PE composites’ char residues (**b**); O1 s XPS spectra of PA66 (**c**), and O1 s XPS spectra of PA66/6% Di−PE (**d**).

The mechanism by which Di−PE could promote char formation of nylon 66 was further explored by FTIR analysis, as shown in Figure 6a,b. It can be seen that with the addition of Di−PE, the characteristic peak of the carboxyl group at 3080 cm^−1^ for the samples PA66/4 wt% Di−PE and PA66/6 wt% gradually decreased. XPS can determine precisely the reaction between a polymer and an additive by the change in chemical bonding. In Figure 6c, the O1 s XPS spectrum of PA66 was divided into two peaks at 532.0 and 531.0 eV, which corresponded to the C=O and COOH bonding states, respectively [30]. Compared with the O1 s XPS spectrum of PA66, the O1 s spectrum of PA66/6 wt% Di−PE was divided into four peaks, and those of the COOH and C=O groups shifted to 531.9 eV and 530.6 eV, respectively [31]. The new peaks at 532.7 and 533.4 eV corresponding to the C-OH and C-O-C bonding states, indicated that the terminal carboxyl group in nylon 66 was blocked, which could lead to a local cross-linked structure, thus helping to promote the formation of a more stable char layer during the combustion process. The FTIR of the char residues also confirmed the above conjecture (Figure 6b). The char residues of PA66 and the PA66/Di−PE composites revealed some typical characteristic peaks at 2918 cm^−1^ and 2856 cm^−1^ attributed to the -CH_2_- stretching vibration. Meanwhile, some new characteristic peaks were observed at 1648, 1531, 1456, 1375, and 723 cm^−1^. The peak at 1648 cm^−1^ corresponded to the stretching vibration of the aromatic structure of the benzene ring, and the characteristic peak at 723 cm^−1^ was attributed to the Carom-H rocking [32], showing that Di−PE could promote the formation in PA66 of a more stable benzene-containing char layer [32,33]. In addition, it was observed that the characteristic peak at 1531 cm^−1^ (-CONH-) of PA66 was not observed for the char residues, while the char residues of the PA66/Di−PE composites retain a visible characteristic peak here, proving that Di−PE caused the incomplete degradation of PA66 [34]. The peaks at 1456 and 1375 cm^−1^ correspond to the -CN- stretching vibration [35,36,37]. Char residues with such structures were more conducive to the improvement of the flame-retardant properties.

**Figure 7:**
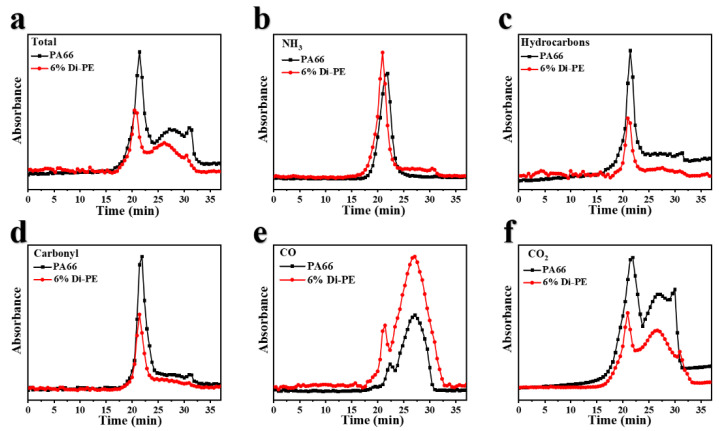
Relationship between absorbance and time for typical evolved products from pure PA66 and PA66/6 wt% Di−PE: (**a**) total absorption; (**b**) NH_3_; (**c**) hydrocarbons; (**d**) carbonyl compounds; (**e**) CO; (**f**) CO_2_.

TG-IR is used to characterize volatile components during heating in the air atmosphere. It could help us to investigate the flame-retardance mechanism of Di−PE in the gas phase. It can be observed in Figure 7a that the pyrolysis behavior of PA66/6 wt% Di−PE shifted to an earlier time, and the absorption intensity of the volatilized components decreased significantly compared to pure PA66, which is in accordance with the CCT results. The characteristic peaks of major pyrolysis volatiles, such as NH_3_ (930–960 cm^−1^), hydrocarbons with the C-H functionality (2850–2950 cm^−1^), carbonyl compounds (aldehydes, ketones, carboxylic acids, 1633–1839 cm^−1^), CO (2150–2180 cm^−1^), and CO_2_ (2300–2400 cm^−1^), were further investigated [38]. As depicted in Figure 7b, the absorption intensity of NH_3_ for PA66/6 wt% Di−PE slightly increased compared to that of pure PA66, indicating a possible dilution of the flammable gas and oxygen, thus inhibiting gas combustion in the gas phase [39]. Furthermore, it was seen that the release of hydrocarbons (Figure 7c) and carbonyl compounds (Figure 7d) during the thermal decomposition of PA66/6 wt% Di−PE decreased significantly compared to that observed for pure PA66, which is beneficial for char formation and ensures the presence of less fuel into the fire zone [40]. In addition, the increased absorption intensity of CO and the decreased absorption intensity of CO_2_ confirmed that Di−PE promoted the incomplete combustion of the PA66/Di−PE composites, which is in accordance with the CCT results. All these observations confirmed that Di−PE also has a positive effect in the gas phase during the combustion process.

### 3.4. Tensile Properties of the PA66/Di−PE Composites

**Figure 8:**
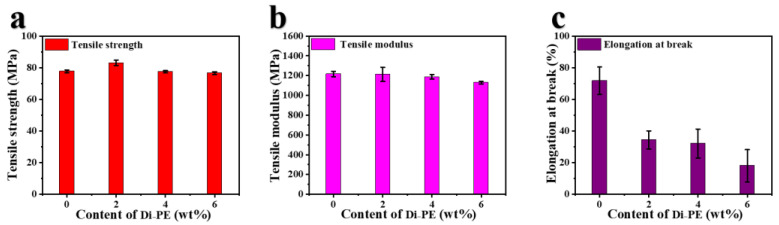
Tensile strength (**a**); tensile modulus (**b**), and elongation at break (**c**) of PA66 and the PA66/Di−PE composites samples.

The tensile properties of pure PA66 and the PA66/Di−PE composite are shown in Figure 8 and Table 5. It can be seen that the addition of Di−PE had no significant effect on the tensile strength and the tensile modulus of PA66, but significantly reduced its elongation at break, from 71.7 ± 8.7% for pure PA66 to 18.0 ± 10.2% for PA66/6 wt% Di−PE. This could be due to the partial cross-linking between the hydroxyl group of Di−PE and the terminal carboxyl group of PA66.

### 3.5. Crystallization Behavior of PA66 and the PA66/Di−PE Composites

**Figure 9:**
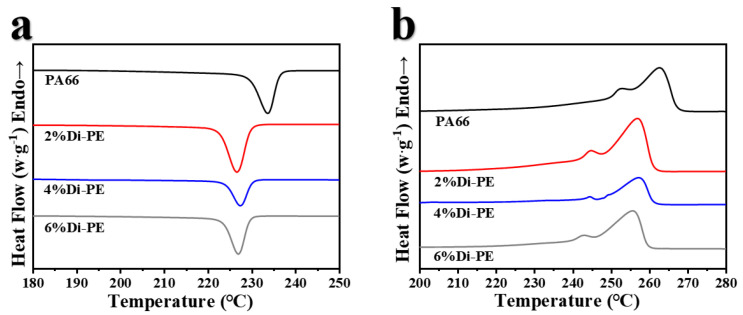
DSC cooling curves (**a**) and heating curves (**b**) of PA66 and the PA66/Di−PE composites.

The influence of Di−PE on the crystallization behavior of the PA66/Di−PE composites was investigated by DSC. The DSC cooling and second heating curves of PA66 and the PA66/Di−PE composites are shown in Figure 9. As clearly observed, the PA66/Di−PE composites showed similar heating and cooling curves to those of pure PA66, indicating that the incorporation of Di−PE had little influence on the crystal form of the PA66/Di−PE composites. However, the crystallization temperature of the PA66/Di−PE composites decreased by nearly 7 °C compared with that of pure PA66 (Figure 9a), which was probably due to the fact that the incorporation of Di−PE hindered the movement of the PA66 chains and made the crystal structure less perfect, leading to a decrease in the melting point (Figure 9b). Nevertheless, the crystallinity of the PA66/Di−PE composite was slightly improved (Table 6).

### 3.6. Mechanical Properties of PA66 and PA66/Di−PE Fibers

**Figure 10:**
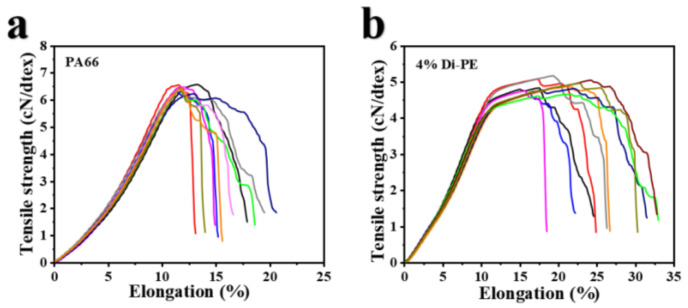
Tensile curves for PA66 fibers (**a**) and PA66/4 wt% Di−PE fibers (**b**).

Thanks to the excellent melt processing performance and high thermal stability of the PA66/Di−PE composites, PA66/Di−PE fibers could be obtained via melt spinning. Figure 10 shows the tensile strength test process of PA66 fibers and the PA66/Di−PE composite fibers, and Table 7 shows the mechanical properties of PA66 and PA66/4 wt% Di−PE fibers. It is clearly seen that, although the tensile strength and modulus of the flame-retardant fiber were slightly decreased, they could still reach 5.7 cN/dtex and thus still meet the requirements of most applications.

### 3.7. Burning Behavior of PA66 and PA66/Di−PE Composites Fabric

**Figure 11:**
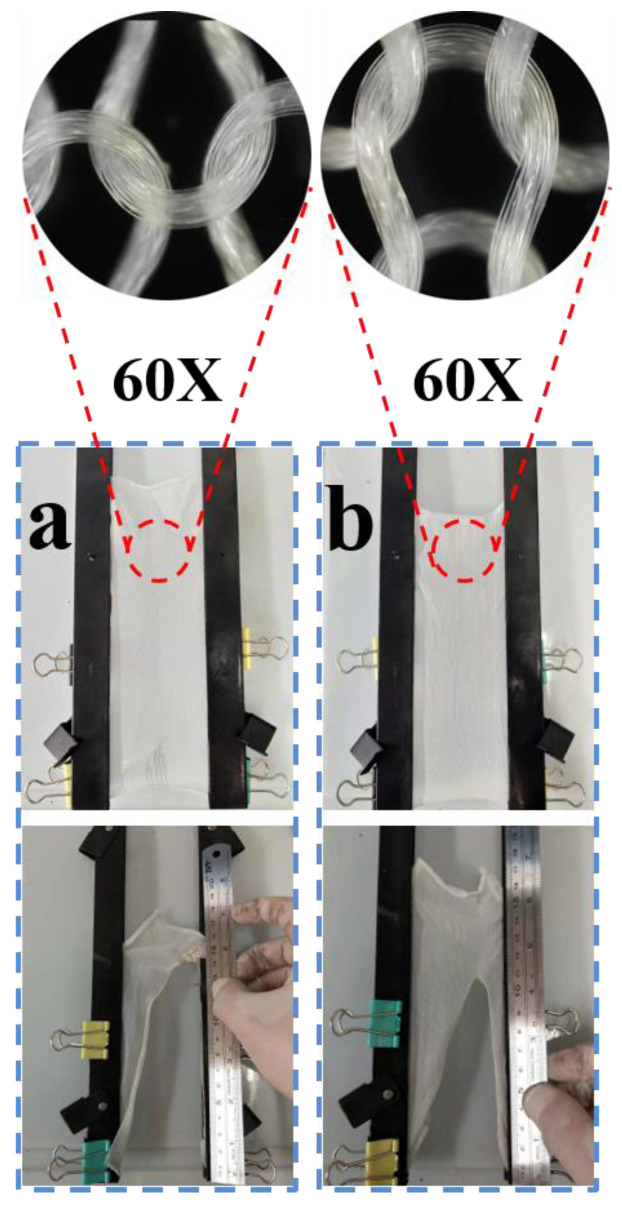
Photographs of PA66 (**a**) and PA66/4 wt% Di−PE (**b**) before and after the vertical burning test of the fabric samples.

In addition to the mechanical properties, the maintenance of the flame-retardant properties of fibers is very important. Figure 11a,b shows the digital photos of knitted PA66 and PA66 4 wt% Di−PE fabrics, which were used for LOI testing. As can be seen, there was no difference in color, indicating that the addition of Di−PE had little effect on the appearance of the fibers. More excitingly, the LOI of the PA66/4 wt% Di−PE fabrics was still as high as 28.6%, and a UL-94 V-0 rating could also be reached. In addition, the flame-retardant fibers could self-extinguish within 3 s, with an average damaged length of 11.1 cm, largely better than that measured for pure PA66 fabrics (Table 8). All these results indicated that Di−PE is a very effective flame retardant for PA66 and has little effect on the fiber forming process; more valuably, the flame-retardant properties of the PA66/Di−PE composites can be highly maintained in the fibers.

## 4. Conclusions

Flame retardant PA66/Di−PE composites and fibers were prepared by blending and the melt spinning technology. It was found that Di−PE could significantly improve the flame-retardant properties of PA66 through the end-group blocking effect. The PA66/6 wt% Di−PE composite achieved the most outstanding LOI value of 29.4% and passed the V-0 rating of UL-94. The CCT results of PA66/6 wt% Di−PE showed that the PHRR and THR were reduced by 47.3% and 47.8%, respectively, compared to those of pure PA66. The FRI of the PA66/4 wt% Di−PE and PA66/6 wt% Di−PE composites reached 1.53 and 2.42, respectively. Moreover, the addition of Di−PE was confirmed to lead to the formation of a continuous and compact char layer, which could cut off the transfer of heat, combustible gas, and oxygen, as well as decrease the release of total gas and flammable hydrocarbons and increase the release of incombustible gas NH_3_. More importantly, the flame retardant PA66/4 wt% Di−PE composites could be made into fibers by melt spinning. The LOI, after-flame time, and damaged length of the PA66/4 wt% Di−PE fabric were significantly improved compared with those of pure PA66, and the absorbent cotton was not ignited. This work provides an outstanding industrial production strategy for fabricating flame-retardant PA66 plastics and fibers and shows that Di−PE has a great potential to reinforce the flame retardancy of PA66.

## Figures and Tables

**Table 1:** Melt spinning parameters for PA66 and the PA66/Di−PE composites.

Melt Spinning Parameters	Value
Barrel temperatures of zone one (°C)	280
Barrel temperatures of zone two (°C)	300
Barrel temperatures of zone three (°C)	295
Barrel temperatures of zone four (°C)	295
Temperature of transfer pipe (°C)	300
Temperature of metering pump (°C)	300
Temperature of spinneret (°C)	300
Head pressure of extruder (bar)	50
Speed (r/min)	22
Speed of metering pump (r/min)	25
Number of spinneret orifices	36
Diameter of spinneret orifices (mm)	0.3
L/D of spinneret orifices	3.0
Take-up velocity, VL (m/min)	800
Distance from the spinneret to the first roller (m)	3.5

**Table 2:** TGA results for PA66 and the PA66/Di−PE composites under air.

Specimen	T_5%_ (°C)	T_max_ (°C)	Char Yields at 600 °C (wt%)	Char Yields at 700 °C (wt%)
Pure PA66	396.3	477.7	2.05	0
PA66/2 wt% Di−PE	376.8	465.7	1.93	0
PA66/4 wt% Di−PE	362.8	473.8	3.82	0.53
PA66/6 wt% Di−PE	352.7	467.0	3.60	1.31

**Table 3:** The results of LOI and UL-94 tests of PA66 and PA66/Di−PE composite.

Sample	PA66	Di−PE	LOI (%)	UL-94 Rating
1	100	0	23.5	V-2
2	98	2	28.8	V-2
3	96	4	29.3	V-0
4	94	6	29.4	V-0

**Table 4:** Relevant parameters for PA66 and FRPA66 obtained from the calorimetric test.

Samples	PA66	PA66/2 wt% Di−PE	PA66/4 wt% Di−PE	PA66/6 wt% Di−PE
TTI (s)	75	61	51	50
PHRR (kW/m^2^)	1059.8	1130.1	685.4	558.7
av-HRR (kW/m^2^)	359.6	311.3	262.0	112.8
THR (MJ/m^2^)	115.1	121.4	78.9	60.1
EHC (MJ/kg)	30.4	29.7	19.7	22.1
PMLR (g/s)	0.341	0.326	0.307	0.233
av-MLR (g/s)	0.105	0.092	0.098	0.045
COY (kg/kg)	0.016	0.025	0.023	0.022
CO_2_Y (kg/kg)	2.315	2.289	1.194	1.223
PSPR (m^2^/s)	0.103	0.114	0.096	0.058
TSP (m^2^)	8.40	9.22	6.24	4.64
FRI	1	0.72	1.53	2.42

**Table 5:** Tensile properties of PA66 and the PA66/Di−PE composites samples.

Sample	Tensile Strength (MPa)	Tensile Modulus (MPa)	Elongation at Break (%)
PA66	77.7 ± 0.9	1216.3 ± 26.7	71.7 ± 8.7
PA66/2 wt% Di−PE	79.5 ± 1.7	1212.2 ± 72.6	34.4 ± 5.7
PA66/4 wt% Di−PE	77.4 ± 0.7	1187.5 ± 22.9	32.1 ± 9.1
PA66/6 wt% Di−PE	76.6 ± 0.9	1129.7 ± 12.9	18.0 ± 10.2

**Table 6:** DSC data for PA66 and the PA66/Di−PE composites.

Samples	T_c_ (°C)	T_m_ (°C)	H_m_ (J/g)	X_c_ (%)
PA66	233.7	262.7	71.8	31.8
PA66/2 wt% Di−PE	226.7	257.0	80.1	35.4
PA66/4 wt% Di−PE	227.3	257.3	75.5	33.4
PA66/6 wt% Di−PE	227.0	255.7	72.1	31.9

**Table 7:** Tensile properties of PA66 and PA66/Di−PE fibers.

Sample	Linear Density (dtex/36F)	Draft Ratio	Elongation at Break (%)	Tensile Strength (cN/dtex)	Modulus (cN/dtex)
PA66	91.9	4.4	12.0 ± 0.6	6.4 ± 0.1	39.6 ± 2.1
PA66/4 wt% Di−PE	115.2	4.8	16.1 ± 1.8	5.7 ± 0.2	31.7 ± 1.5

**Table 8:** Flame retardance of PA66 and PA66/Di−PE composites fabric samples.

Sample	LOI (%)	After-Flame Time (s)	Damaged Length (cm)	Ignite Absorbent Cotton	Burn Behavior
PA66 fabric	23.1	5.0 ± 0.26	15.6 ± 0.6	yes	Melts, drips, and shrinks away from the flame
PA66/4 wt% Di−PE fabric	28.6	2.0 ± 0.19	11.1 ± 0.6	no	Self-extinguishing within 3 s, minimum damaged length.

## Data Availability

Not applicable.

## Supplementary Materials

The following supporting information can be downloaded at: https://www.mdpi.com/article/10.3390/polym15051183/s1, Figure S1: Pure PA66 and PA66/6% Di-PE composites in muffle furnace at different temperature; Figure S2: Digital photos of Pure PA66 (a), PA66/2% Di-PE (b), PA66/4% Di-PE (c) and PA66/6% Di-PE (d) after LOI test; Table S1: Representative information of PA66 flame retardant modified from recent years [41,42,43,44,45,46,47,48].
