# Supplementary material for: Flame Retardancy and Mechanical Properties of Melt-Spun PA66 Fibers Prepared by End-Group Blocking Technology
Source: Polymers (Basel). 2023 Feb 26;15(5):1183. doi: 10.3390/polym15051183 (PMC10007604; doi:10.3390/polym15051183)
Supplement: Supplementary file 1 [file polymers-15-01183-s001.zip › polymers-2251505-supplementary.pdf]

Flame Retardancy and Mechanical Properties of Melt-Spun PA66 Fibers Prepared by End-Group Blocking Technology

Yanpeng Wu [1], Tonghui Yang [1], Yongchang Cheng [1], Tao Huang [1,*], Bin Yu [1,*], Qilin Wu [1,*], Meifang Zhu [1] and Hao Yu [1]

State Key Laboratory for Modification of Chemical Fibers and Polymer Materials, College of Materials Science and Engineering, Donghua University, Shanghai 201620, P. R. China

* Correspondence: ht@dhu.edu.cn (T. Huang), yubin@dhu.edu.cn (B. Yu), wql@dhu.edu.cn (Q. L. Wu)

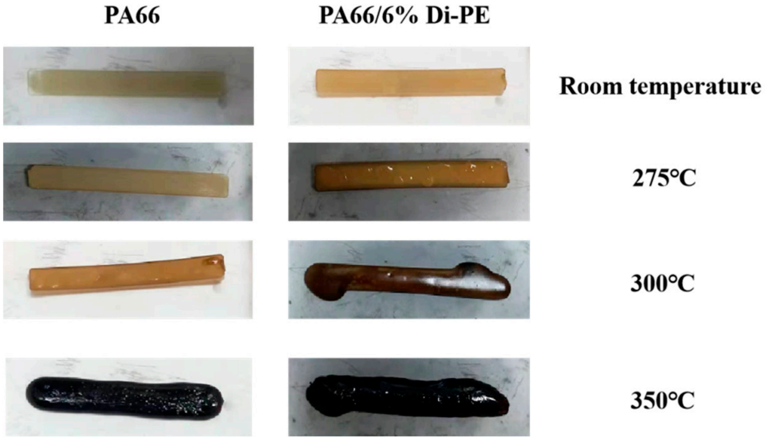

Figure S1 Pure PA66 and PA66/6% Di-PE composites in muffle furnace at different temperature

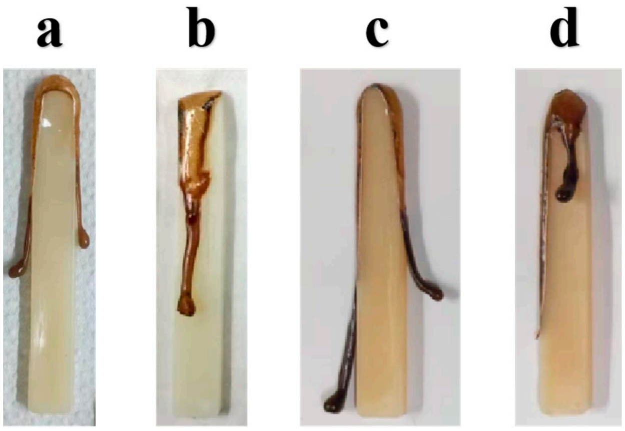

Figure S2 Digital photos of Pure PA66 (a)、PA66/2% Di-PE (b)、PA66/4% Di-PE (c) and PA66/6% Di-PE (d) after LOI test.

Table S1 Representative information of PA66 flame retardant modified from recent years.

Materials	Method	Content	LOI (%)	UL-94	Cone calorimeter power (kW/m^2)	PHRR (kW/m^2)	THR (MJ /m^2)	Reference
PA66-10% MCPO	Copolymerization	10	29.0	V-0	50	429.0	60.2	[1]
PA66-6% CEPPA	Copolymerization	6	28.0	V-0	-	-	-	[2]
PA66-5% DDP-HMDA	Copolymerization	5	33.2	V-0	50	712.0	105.9	[3]
PA66-5% NENP	Copolymerization	5	28.0	V-0	50	432.6	72.9	[4]
PA66-4.5% PDPPD	Copolymerization	4.5	29.0	V-0	50	570.0	80.0	[5]
83PA66/12% ADCP/5% Nano-Silica	Blending	17	35.2	V-0	35	462.2	40.7	[6]
84.5PA66/6% MCA/6% ALPi/2.5% Gr	Blending	14.5	-	V-0		-	-	[7]
90PA66/9.7% ALPi/0.3% MgO	Blending	10	32.8	V-0	50	698.0	98.0	[8]
90PA66/10% ADCP	Blending	10	28.0	V-0	-	-	-	[9]
90PA66/10DT	Blending	10	27.6	-	50	481.0	48.6	[10]
PA66/6% Di-PE	Blending	6	29.4	V-0	50	558.7	60.1	This work

References

1.Lyu, W.; Chen, X.; Li, Y.; Cao, S.; Han, Y., Thermal stability and heat release effect of flame retarded PA66 prepared by end-pieces capping technology. *Composites Part B: Engineering* **2019,** *167*, 34-43.

2.Chen, X.; Xu, D.; Zhang, H.; Feng, X.; Deng, J.; Pan, K., In situ polymerization of flame retardant modification polyamide 6,6 with 2-carboxy ethyl (phenyl) phosphinic acid. *Journal of Applied Polymer Science* **2020,** *137* (20), 48687.

3.Li, Y.; Liu, K.; Zhang, J.; Xiao, R., Preparation and characterizations of inherent flame retarded polyamide 66 containing the phosphorus linking pendent group. *Polymers for Advanced Technologies* **2018,** *29* (2), 951-960.

4.Lyu, W.; Cui, Y.; Zhang, X.; Yuan, J.; Zhang, W., Thermal stability, flame retardance, and mechanical properties of polyamide 66 modified by a nitrogen–phosphorous reacting flame retardant. *Journal of Applied Polymer Science* **2016,** *133* (24), 43538.

5.Lyu, W.; Cui, Y.; Zhang, X.; Yuan, J.; Zhang, W., Fire and thermal properties of PA 66 resin treated with poly-N-aniline-phenyl phosphamide as a flame retardant. *Fire and Materials* **2017,** *41* (4), 349-361.

6.Zhang, H.; Lu, J.; Yang, H.; Yang, H.; Lang, J.; Zhang, Q., Synergistic flame-retardant mechanism of dicyclohexenyl aluminum hypophosphite and nano-silica. *Polymers* **2019,** *11* (7), 1211.

7.Luo, D.; Liu, Y.; Shi, Y.; Wang, Q., The properties of flame retardant and heat conduction polyamide 66 based on melamine cyanurate/aluminum diethylphosphinate/graphene. *Journal of Polymer Research* **2019,** *26* (9), 216.

8.Zhan, Z.; Shi, J.; Zhang, Y.; Zhang, Y.; Zhang, B.; Liu, W., The study on flame retardancy synergetic mechanism of magnesium oxide for PA66/AlPi composite. *Materials Research Express* **2019,** *6* (11), 115317.

9.Zhang, H.; Lu, J.; Yang, H.; Lang, J.; Yang, H., Comparative study on the flame-retardant properties and mechanical properties of PA66 with different dicyclohexyl hypophosphite acid metal salts. *Polymers* **2019,** *11* (12), 1956.

10.Guo, S.; Bao, M.; Ni, X., The synthesis of meltable and highly thermostable triazine-DOPO flame retardant and its application in PA66. *Polymers for Advanced Technologies* **2021,** *32* (2), 815-828.
